# Unique Treatment of Atrial Fibrillation: Simultaneous Dual Direct-Current Cardioversion in Morbidly Obese Patients

**DOI:** 10.7759/cureus.80837

**Published:** 2025-03-19

**Authors:** Bo Peng, Shad Sommerville, Allyson Tragesser, Yong G Peng

**Affiliations:** 1 Department of Anesthesiology, University of Florida College of Medicine, Gainesville, USA

**Keywords:** anticoagulation, atrial fibrillation, dual direct-current cardioversion, morbid obesity, pharmacological and nonpharmacological intervention, rate control, rhythm conversion

## Abstract

Atrial fibrillation (AFib) is a prevalent arrhythmia associated with substantial health complications and economic strain on the health care system in the United States. Obesity is a challenging comorbidity to manage in this scenario, as the incidence of AFib rises alongside increasing body mass index (BMI). Furthermore, obesity presents several challenges in applying traditional treatment modalities for AFib, such as pharmacological options, ablation, and direct-current cardioversion (DCCV). Currently, there is no specific regimen for treating AFib for morbidly obese patients. This case report describes and discusses a unique treatment option for a morbidly obese patient with poorly controlled AFib.

A 43-year-old male with several comorbidities including morbid obesity with a BMI of 87 kg/m^2^ presented with AFib in the setting of sepsis and bacteremia. The patient was treated with increasing doses of metoprolol without effective control of his AFib and had previously received amiodarone as well without appropriate response. DCCV was performed under deep sedation using dexmedetomidine and propofol infusions with careful attention to maintaining spontaneous ventilation. AFib was successfully converted to normal sinus rhythm using dual biphasic DCCV (each at 200 J).

Pharmacological treatment of AFib can be challenging in the obese population due to medications and traditional cardioversion being less effective with increasing BMI. The anesthetic plan was of important consideration in this case, given his BMI of 87 kg/m^2^ and the risk for obstruction and potential airway compromise. Nasal continuous positive airway pressure and the use of dexmedetomidine supported airway patency and maintained spontaneous respirations while ensuring adequate depth of anesthesia. Morbidly obese patients likely require increased energy for cardioversion; thus, we describe the effectiveness of using simultaneous dual DCCV with two sets of pads in this patient after an unsuccessful single biphasic shock with 200 J.

## Introduction

Atrial fibrillation (AFib) is the most common cardiac arrhythmia. In 2016, the Global Burden of Disease project estimated over 46.3 million reported cases worldwide [1]. AFib is associated with significant morbidity, mortality, and financial burden on the health care system in the United States [1]. Numerous risk factors have been implicated as causes of AFib, including valvular disease, coronary artery disease, diabetes, kidney disease, smoking, poor nutritional status, and sepsis. However, the relationship between obesity and AFib has become a recent focus of research. A meta-analysis suggested that the incidence of AFib increases by 29% for every five numerical increases in BMI, indicating an association between BMI and AFib [2]. This has become a real concern with the rising prevalence of obesity in recent decades [1]. The treatment of obese patients with AFib is challenging and less than ideal, often leading to persistent AFib and subsequent morbidities [3]. Many conventional therapies, such as the utilization of antiarrhythmic medications, anticoagulation, and ablation, are less effective in patients with severe obesity [4]. Despite these outcomes, there is still a lack of any specific therapeutic options for treating AFib in morbidly obese patients.

One common treatment for AFib is the use of direct-current cardioversion (DCCV). It has been reported that the routine use of cardioversion was less effective in obese patients [5]. In this report, we describe a case of a morbidly obese patient with a BMI of 87 kg/m^2^ who was treated with failed single DCCV, followed by successful simultaneous dual DCCV to a normal sinus rhythm. We also highlight the anesthetic approach used to maintain spontaneous ventilation and expeditious recovery in a morbidly obese patient who is at high risk for airway complications.

## Case presentation

A 43-year-old man presented with a history of hypertension, diabetes, chronic kidney disease, heart failure with preserved ejection fraction (HFpEF), obstructive sleep apnea, morbid obesity (BMI 87 kg/m^2^), and new-onset AFib. His weight and height at the time of presentation were 567 lb 11 oz (257.5 kg) and 5 feet 10 inches, respectively. The patient was initially admitted to the hospital secondary to shortness of breath, cough, fever, diarrhea, decreased urine output, and elevated lactic acid with a diagnosis of sepsis. Blood cultures were positive for *Streptococcus pyogenes*, which was treated with vancomycin. An electrocardiogram revealed AFib/atrial flutter with rapid ventricular response. The patient had previously failed to respond to conventional antiarrhythmic medications metoprolol and amiodarone. Due to his history of HFpEF, cardiology consultation led to an increased dose of metoprolol without sufficient rate control during his seven-day course of antibiotics. The CHA2DS2-VASC score was reported as 3.

After interdisciplinary discussion, the decision was made to perform the transesophageal echocardiography (TEE) and follow-up with DCCV in the operating room rather than the cardiology suite. This plan was with specific consideration of the patient’s large body habitus and potential for airway compromise during the procedure. Deep sedation was planned to preserve the patient’s spontaneous respiratory status, avoid a possible difficult airway, provide comfort with tolerating the cardioversion procedure, and aim for an expeditious recovery. In the operating room, American Society of Anesthesiologists standard monitors and a bispectral index (BIS) monitor were placed to assist with titration and level of sedation throughout the case with a target level around 40 to 60 during cardioversion. A SuperNO2VA™ (Vyaire Medical, Mettawa, IL, USA) was placed to support spontaneous breathing and to avoid airway obstruction.

**Figure 1:**
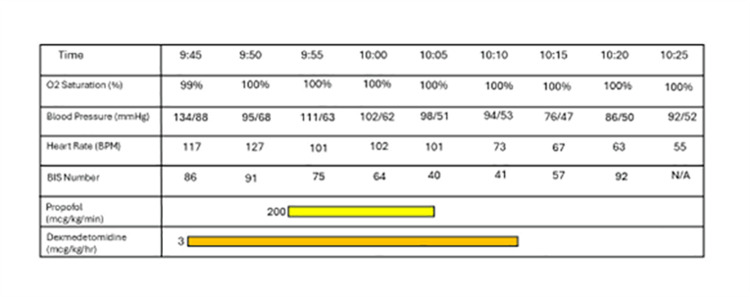
Summary of patient’s anesthesia record.

**Figure 2:**
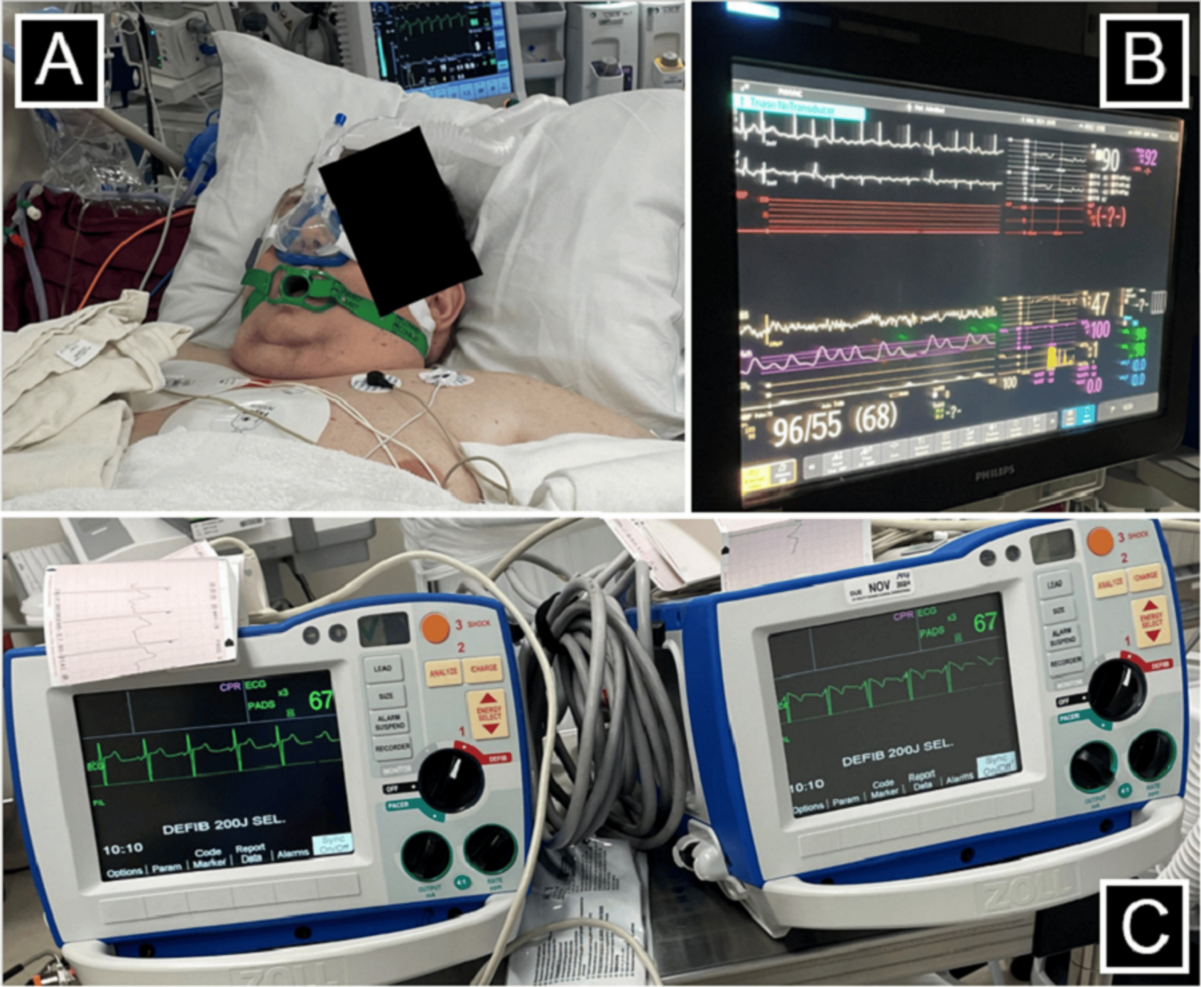
Patient’s treatment setup. Figure 2A shows the SuperNO2VA™ placement and precautions taken during the patient’s treatment. Figure 2B shows the hemodynamic AFib prior to cardioversion. Figure 2C shows the hemodynamic rhythm following treatment using dual synchronized cardioversion using biphasic current with 200 J x2 simultaneous discharge. AFib, atrial fibrillation

**Figure 3:**
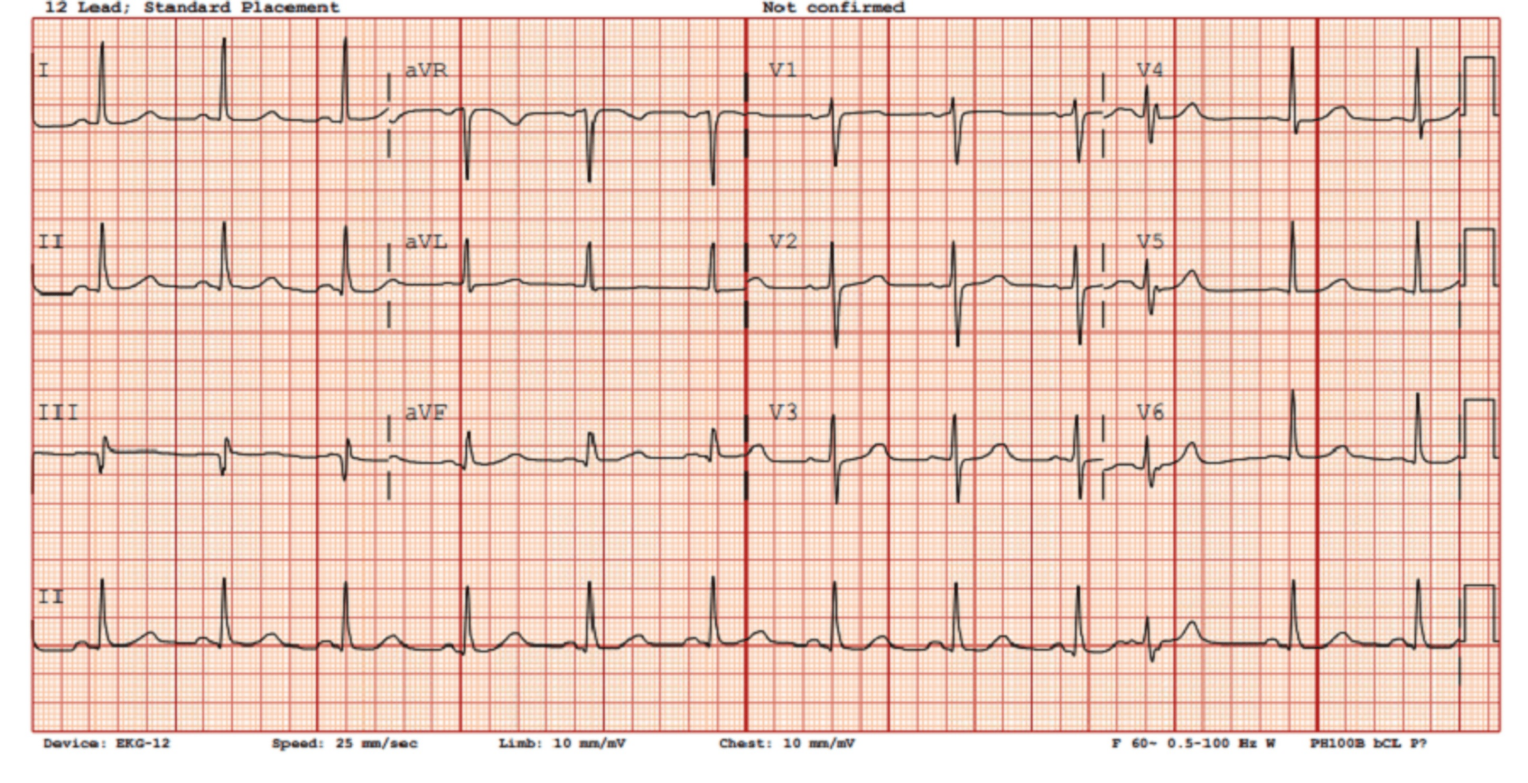
Patient’s 12-lead electrocardiogram immediately after cardioversion demonstrating normal sinus rhythm.

The patient was sedated with dexmedetomidine and propofol infusion based on 100 kg of body weight (estimate of lean body weight). The dexmedetomidine infusion was programmed to 3 mcg/kg/hour and run for approximately 9 minutes prior to the addition of the propofol infusion at 200 mcg/kg/minute based on 100 kg of body weight. After confirming that the patient was adequately sedated, the TEE probe was lubricated with lidocaine jelly and placed without significant resistance, and the patient tolerated the manipulation well. The TEE revealed no evidence of thrombus in the left atrial appendage. Two sets of pads were placed: one set in the anterior-posterior position and the second set in the right-posterior to the left-lateral position. Initially, single biphasic synchronized cardioversion at 200 J was performed without resolution of AFib. Therefore, a second attempt using dual biphasic synchronized cardioversion at 200 J x2 was performed with simultaneous discharge of both machines, which led to the successful conversion of AFib to normal sinus rhythm at 66 beats per minute. Overall, the patient tolerated the procedure well without airway obstruction or hemodynamic instability. A summary of the patient’s anesthesia record is shown in Figure 1. The patient’s treatment setup is shown in Figure 2. The patient’s electrocardiogram immediately after cardioversion demonstrating normal sinus rhythm is shown in Figure 3. The patient remained in normal sinus rhythm over two months after DCCV; however, he was unfortunately lost to follow-up afterwards.

## Discussion

Traditional therapeutic options for AFib can be classified into two categories: pharmacological and nonpharmacological treatments. Medical treatments usually consist of anticoagulation therapy for patients with a CHA2DS2-VASC score greater than 2, rate control, and rhythm conversion. Anticoagulation is given as a routine treatment for patients in whom conversion of AFib to sinus rhythm fails. Procedural intervention for AFib includes left atrial appendage occlusion, cardioversion, catheter ablation, and maze procedure [6]. Since our patient was morbidly obese with numerous concomitant medical conditions, pharmacological treatment proved to be ineffective, and there was concern for unpredictable side effects if this treatment continued [4]. It has been reported that there was no clinical difference in outcomes between strict and lenient rate control in patients with AFib. Patients were randomized to the lenient group with rates of <110 beats per minute versus the strict group with rates of <80 beats per minute and showed similar cardiovascular outcomes [7]. Beta-blockers are reported to be less effective in patients with obesity, diabetes, and HFpEF, and, in fact, may cause additional risks due to potential bradyarrhythmias and heart block [8]. The pathophysiology of this patient population is described as an inflammatory expansion of adipose tissue in the left atrium, which can lead to electromechanical remodeling [8]. In obese patients, rate control medication can often fail as the first line of therapy, which was exhibited in our patient. It is crucial to search for an additional therapeutic option in this patient population.

Another important point to consider is the etiology of this patient’s AFib. Although it is likely that obesity was a significant contributing factor, the patient's initial diagnosis of sepsis could also be the reason for the development of AFib. Meta-analysis reveals that up to 8% of patients with sepsis develop new-onset AFib [9]. Despite the patient being treated with a 2-week course of antibiotics for his sepsis, his AFib persisted, requiring intervention. Given the other comorbidities with this patient, including sepsis, obesity, diabetes, chronic kidney disease, and heart failure, it was imperative to convert his AFib into sinus rhythm.

Performing TEE and cardioversion procedures for obese patients under deep sedation or general anesthesia remains debatable. Generally, there is no significant difference regarding safety profiles between general anesthesia and sedation techniques. However, it was noted that 0.3% of patients under general anesthesia recalled the intraoperative event versus 2% in the deep sedation group [10]. Patients with morbid obesity have many potential issues with their airways, including increased adipose tissue in the pharynx, larger tongue, and adenoid tissue, all making obstruction more likely. The patient's high BMI, increased neck circumference, and Mallampati score of 4 all made intubation and mask ventilation more likely to be difficult. Cook et al. [11] noted that the chances were twice as likely for obese patients to develop airway problems, including challenges with face masking and tracheal intubations. In addition, a 2019 study performed by Kilic et al. [12] showed that deep sedation in the obese population was safe and effective for endoscopic procedures. As a similar procedure, the deep sedation approach seemed like a safe and effective option for this case.

There is, however, a rather thin line between deep sedation and general anesthesia. In some incidences, conversion of sedation to general anesthesia is required. Despite this, one study showed that only 0.5% of monitored anesthesia care sedation cases required conversion to general anesthesia, with the majority of those cases including certain surgical specialties such as oral maxillofacial surgery, otorhinolaryngology, and orthopedic surgery [13]. Due to the heightened concern for airway challenges in our patient, it was deemed more appropriate to perform his cardioversion in the operating room setting. Preprocedural planning and discussion included many characteristics of the difficult airway algorithm. We ensured expedient access to a supraglottic airway device, video laryngoscopy, and fiberoptic bronchoscopes in the event that we were unable to maintain the patient’s breathing spontaneously. Additionally, we placed a SuperNO2VA™ device on our patient, allowing for continuous positive airway pressure (CPAP) throughout the procedure. Nasal CPAP attempts to minimize airway obstruction and maintain airway patency, particularly in those at risk for obstruction and a history of obstructive sleep apnea or obesity. A published case study by Kozinn et al. [14] described using the CPAP method for a patient with a BMI of 47.2 kg/m^2^ requiring deep sedation for TEE and cardioversion. They reported maintaining an oxygen saturation (SpO_2_) of 99% for the duration of the procedure. Similarly, we achieved an SpO_2_ level of 99% to 100% for our patient (BMI 87 kg/m^2^) throughout the duration of the procedure.

Our anesthetic plan with respect to pharmacological decisions also prioritized the ability of the patient to preserve their own spontaneous respiratory status while maintaining appropriate patient comfort. Preoperatively, the patient was administered 2 mg of midazolam. This short-acting benzodiazepine with a fused imidazole ring is largely used for its anxiolytic and amnestic properties [15]. Often utilized for its respiratory preserving characteristic, a dexmedetomidine infusion was initiated at 3 mcg/kg/hour. Dexmedetomidine is a potent and selective alpha-2 agonist that has anxiolytic, sedative, and analgesic properties. It was first approved for use in nonintubated patients for surgical/procedural sedation in 2008 in the United States and continues to be a resourceful medication with its minimal effects on respiration along with its rapid distribution half-life and quick onset of action [16]. Shortly after sedation initiation with dexmedetomidine, a BIS monitor was placed and connected. After 9 minutes of the dexmedetomidine infusion, the BIS monitor displayed a numerical value of 91, and the patient remained aware and arousable. A propofol infusion was then initiated at 200 mcg/kg/minute in addition to the dexmedetomidine infusion. The major sedative effect of propofol occurs via the enhancement of γ-aminobutyric acid (GABA) through the GABAA receptor. It is a highly lipid-soluble drug and is emulsified in a blend of soybean oil, glycerol, and purified egg phospholipids [17]. Consequently, propofol readily crosses lipid bilayers and has a fast-onset, fast-off effect with the rapid termination of its hypnotic effect largely attributable to redistribution into inactive tissues such as muscle and adipose tissue. Despite this rapid distribution into adipose tissue, it is important to remember that it is typically advised to administer dosages based on lean body weight as opposed to total body weight, which can lead to overdosing and hemodynamic instability, particularly in the morbidly obese patient population. Thus, our initial dose reflects an acceptable infusion rate for a relatively quick and efficient procedure. The total propofol infusion time consisted of approximately 15 minutes, with the first 11 minutes displaying a BIS index of >60. Just prior to successful cardioversion and discontinuance of all infusions, the patient had a BIS index with a nadir of 40. Shortly after termination of the intravenous infusions, the patient emerged from sedation and was able to maintain his spontaneous respiratory status throughout the procedure. Afterwards, relatively small amounts of phenylephrine were administered for hemodynamic support while the patient fully recovered from the vasodilatory effects of the anesthesia.

After TEE evaluation confirmed that there was no evidence of thrombosis in the left atrial appendage, the team chose to perform synchronized cardioversion using a single biphasic shock of 200 J without success. The patient subsequently received dual synchronized cardioversion using biphasic 200 J x2 simultaneous discharge, which successfully converted his AFib to sinus rhythm. The rationale for using double amplitude power in cardioversion was because of recent examples of the effectiveness. A trial by Gallagher et al. in 2001 [18] showed that a setting of ≥360 J could achieve cardioversion at a higher efficiency, particularly in AFib, using mono-shock therapy. Multiple trials have investigated the effectiveness of using dual biphasic cardioversion in obese patients. The randomized trial by Aymond et al. [19] reported greater efficacy of dual DCCV in cardioversion for patients with AFib compared to single DCCV (98% vs 86%, respectively). They were able to show that cardioversion failed more often with lower power amplitude, and dual DCCV was a successful rescue each time after an unsuccessful single DCCV. Both of these studies reported minimal adverse effects. We used conventional adult multifunctional Zoll pad placement on our patient compared to the patients in these studies. We again demonstrated that using dual synchronized cardioversion with biphasic energy of 200 J x2 with simultaneous discharge can be successful in converting AFib to sinus rhythm in a patient with a BMI of 87 kg/m^2^. Additional studies are needed to evaluate the utility of dual DCCV in normal-sized patients who are resistant to an initial single DCCV.

A plausible explanation for why dual DCCV could be more effective in patients with obesity is because a critical mass of myocardial tissue must be defibrillated to see the net effects. Zipes et al. [20] showed in a dog model that 75% of ventricular myocardium needed to be depolarized to abolish ventricular fibrillation. Although specific for ventricular fibrillation, it would be reasonable to apply this logic to AFib as well, as it explains why more energy is needed in patients with obesity. Patients with obesity likely have more adipose tissue between the chest wall and myocardium, and thus higher energy current must pass an excessive amount of tissue to reach the myocardium.

## Conclusions

Treating morbidly obese patients with many underlying conditions for AFib warrants multiple aspects of consideration. When conventional pharmacological treatment is not effective due to a lack of response, alternative nonpharmacological intervention should be considered. This case demonstrates that careful planning and coordination within the operating room environment was imperative for treating a morbidly obese patient (BMI 87 kg/m^2^) patient with AFib. The use of SuperNO2VA™ and simultaneous dual DCCV under deep sedation with careful titration of dexmedetomidine and propofol can help mitigate clinical risks. Although this is a single case presentation and cannot be applied to the general population, this case demonstrates a novel approach to converting AFib when conventional therapy is ineffective.
